# Methylphenidate for attention-deficit/hyperactivity disorder in patients with Smith–Magenis syndrome: protocol for a series of N-of-1 trials

**DOI:** 10.1186/s13023-021-02003-z

**Published:** 2021-09-08

**Authors:** A. R. Müller, J. R. Zinkstok, N. N. J. Rommelse, P. M. van de Ven, K. C. B. Roes, F. A. Wijburg, E. de Rooij-Askes, C. Linders, E. Boot, A. M. van Eeghen

**Affiliations:** 1 Advisium, ’s Heeren Loo, Amersfoort, the Netherlands; 2 Department of Pediatrics, Emma Children’s Hospital, Amsterdam University Medical Center, Amsterdam, The Netherlands; 3 Department of Psychiatry and Brain Center, University Medical Center Utrecht, Utrecht, The Netherlands; 4 Karakter, Child and Adolescent Psychiatry, Nijmegen, The Netherlands; 5 Department of Psychiatrics, Radboud University Medical Center, Nijmegen, The Netherlands; 6 Department of Epidemiology and Data Science, Amsterdam University Medical Center, Amsterdam, The Netherlands; 7 Department of Health Evidence, Biostatistics, Radboud University Medical Center, Nijmegen, The Netherlands; 8 Department of Psychiatry and Neuropsychology, Maastricht University, Maastricht, The Netherlands; 9 The Dalglish Family 22Q Clinic, University Health Network, Toronto, ON Canada

**Keywords:** N-of-1, Smith–Magenis syndrome, Methylphenidate, Rare genetic neurodevelopmental disorder, Multiple crossover, ADHD

## Abstract

**Background:**

Smith–Magenis syndrome (SMS) is a rare genetic neurodevelopmental disorder characterized by intellectual disability and severe behavioural and sleep disturbances. Often, patients with SMS are diagnosed with attention-deficit/hyperactivity disorder (ADHD). However, the effectiveness of methylphenidate (MPH), the first-line pharmacological treatment for ADHD, in patients with SMS is unclear. Our objective is to examine the effectiveness of MPH for ADHD symptoms in individuals with SMS, proposing an alternative trial design as traditional randomized controlled trials are complex in these rare and heterogeneous patient populations.

**Methods and analysis:**

We will initiate an N-of-1 series of double-blind randomized and placebo-controlled multiple crossover trials in six patients aged ≥ 6 years with a genetically confirmed SMS diagnosis and a multidisciplinary established ADHD diagnosis, according to a power analysis based on a summary measures analysis of the treatment effect. Each N-of-1 trial consists of a baseline period, dose titration phase, three cycles each including randomized intervention, placebo and washout periods, and follow-up. The intervention includes twice daily MPH (doses based on age and body weight). The primary outcome measure will be the subscale hyperactivity/inattention of the Strengths and Difficulties Questionnaire (SDQ), rated daily. Secondary outcome measures are the shortened version of the Emotion Dysregulation Inventory (EDI) reactivity index, Goal Attainment Scaling (GAS), and the personal questionnaire (PQ). Statistical analysis will include a mixed model analysis. All subjects will receive an assessment of their individual treatment effect and data will be aggregated to investigate the effectiveness of MPH for ADHD in SMS at a population level.

**Conclusions:**

This study will provide information on the effectiveness of MPH for ADHD in SMS, incorporating personalized outcome measures. This protocol presents the first properly powered N-of-1 study in a rare genetic neurodevelopmental disorder, providing a much-needed bridge between science and practice to optimize evidence-based and personalized care.

***Trial registration *:**

This study is registered in the Netherlands Trial Register (NTR9125).

## Highlights of the study protocol

- Innovative trial design combining collection of scientific data with personalized care, providing a much-needed bridge between practice and science.
- Evidence-based treatment of ADHD symptoms in Smith–Magenis syndrome.
- The first adequately powered series of randomized, double-blind, placebo-controlled N-of-1 trials for a rare genetic neurodevelopmental disorder.
- Exploring patient-centered outcome measures addressing relevant goals of the patient.

## Background

Smith–Magenis syndrome (SMS) is a rare genetic neurodevelopmental disorder with an estimated prevalence of 1:15.000–25.000 births [1]. SMS is caused by a deletion on chromosome 17 (17p11.2) or a pathogenic mutation in the *RAI1* gene located within this region. Most of the SMS manifestations are due to haploinsufficiency of *RAI1* and thought to be modified by other genes in the 17p11.2 region [2–4]. Manifestations are variable and include intellectual disability (ID), severe sleep disturbances and psychiatric comorbidity such as autism spectrum disorders (ASD), attention-deficit-hyperactivity disorder (ADHD) [5–7]. Typical behavioural manifestations include problems with emotion dysregulation, self-injurious behaviour and aggressive or stereotypical behaviour, posing a great burden on patients and caregivers [8].

Treatment of the behavioural manifestations in SMS is complex due to the genetic heterogeneity, clinical variability and severity of symptoms [4, 9]. Traditionally, treatment is focused on appropriate management of sleeping pattern, concomitant somatic comorbidities, psycho-education and professional guidance for parents and caregivers aimed at symptom reduction and optimizing quality of life of both the patient and their family [10–13]. Often, this does not suffice, resulting in the prescription of psychotropic drugs in the vast majority of children and adults with SMS, including stimulants, antidepressants, antipsychotics, mood stabilizers, alfa2 agonists, sleep aids, and benzodiazepines [14].

For idiopathic ADHD, methylphenidate (MPH) is well-established as first-line treatment with high efficacy and tolerability compared to other psychotropic drugs [15–17]. However, for ADHD in genetic neurodevelopmental disorders such as SMS more information is necessary as there is increasing evidence for differential treatment response and tolerability [14, 18, 19]. Also, polypharmacy is a clinical pitfall in patients with complex psychiatric disorders and ID, leading to iatrogenic comorbidity [20]. Therefore, disorder-specific studies are needed to provide information about the effectiveness of MPH for ADHD. Considering the heterogeneity of the patient population and need for relevancy of interventions, personalized outcome measures are needed to enable measurement of clinically important changes. Such a personalized methodological approach has the potential of maximizing treatment adherence that is both patient-centered and evidence-based [21–23].

### Rationale for N-of-1 design

Trials in rare genetic neurodevelopmental disorders such as SMS pose specific challenges due to comorbidities and rarity of conditions [24, 25]. Single-case experimental designs (SCEDs) provide an alternative to traditional parallel group randomized controlled trials (RCTs). Of SCEDs, the N-of-1 methodology provides the most rigorous evidence for treatment decisions at an individual level as replication is key for confirmation of causality. N-of-1 studies are randomized, controlled, multiple cross-over trials within individual patients [26, 27] and enhance precision when treatment effects are heterogeneous between individuals [28, 29]. Aggregating the results of several N-of-1 trials potentially yields treatment effect estimates that may be generalized at population level and may be as robust as traditional RCTs [30]. In particular, patients with rare disorders require individualized treatment interventions and outcomes due to their heterogeneity and vulnerability, which is facilitated by N-of-1 designs and consistent with the movement towards personalized care, providing a much needed bridge between practice and science [21].

### Objectives

The main objective is to study the effectiveness of MPH for ADHD symptoms in individuals with SMS. Secondary objectives include assessment of the effect of MPH on emotion dysregulation, personalized goals that are specific and important to the patient, and side effects. To do this, we will perform a series of N-of-1 trials as these provide an excellent approach to study effectiveness of MPH on ADHD in SMS, given: (1) the chronic and relatively stable clinical course of ADHD, and (2) the rapid onset and termination of action of MPH [31].

## Methods

### Study design

We used the Standard Protocol Items: Recommendations for Interventional Trials (SPIRIT) extension for N-of-1 trials (SPENT) checklist that is aligned with the CONSORT (consolidated reporting items for trials) extension for N-of-1 trials (CENT) for developing this N-of-1 protocol [29].

**Fig. 1:**
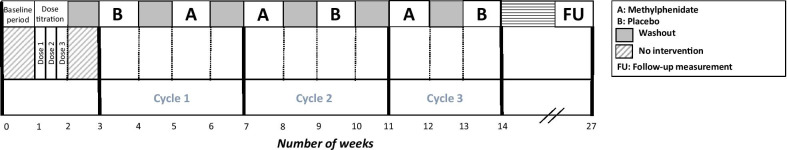
Study design

The study will consist of a series of N-of-1 trials followed by an optional open-label extension phase. Each trial is randomized, placebo-controlled, and double-blinded with multiple crossovers within a single patient. The trial consists of a baseline period, dose titration phase, and three cycles each consisting of one period of MPH treatment and one period of placebo treatment, both followed by a one-week washout period (Fig. 1). Despite the fact that a one-day washout would suffice biologically, we chose one-week washouts to account for prolonged psychological effects that may occur. The order of the treatment periods will be randomized. Thus, each N-of-1 trial will last 14 weeks with an additional follow-up measurement three months after completion of the N-of-1 trial.

#### Protocol development and patient engagement

Collaboration with the Dutch SMS patient advocacy organization, caregivers of patients and clinical experts played a large role in defining knowledge and care gaps, prioritizing the treatment study, development of the current protocol and selecting outcome measures. We addressed specific difficulties for conducting this study, including concerns related to caregiver burden and patient burden of participation, and issues for recruitment and retention.

### Outcome measures

The primary outcome is the change on the hyperactivity/inattention subscale of the Strengths and Difficulties Questionnaire (SDQ) during active interventional periods. Secondary outcome measures are the shortened version of the Emotion Dysregulation Inventory (EDI) reactivity index [32], Goal Attainment Scaling (GAS) [33] and the personal questionnaire (PQ) [34]. Also, (the number of) side effects determined by the side effects checklist of MPH will be recorded.

#### Rationale for outcome measures

The SDQ subscale and the shortened version of the EDI have both been psychometrically considered as valid tools to measure behavior of people with ID and applicable to both children and adults [32, 35, 36]. Specifically, the SDQ was found to be a valid outcome measure for children with ADHD symptoms and showed preliminary results of validation for children with ID [37, 38]. EDI was created using methods developed by the Patient-Reported Outcomes Measurement Information System (PROMIS) and validated as an efficient and sensitive method to measure emotion dysregulation in youth with ASD of any level of cognitive or verbal ability [32, 36]. The EDI will serve as a generalization measure that is defined as an outcome closely or more distally related to the target behavior, and is used to evaluate transfer effects of the intervention to a broader domain of functioning [39]. For instance, it could be the same behavior but in another setting, such as inattention at school and at home, or interventional effects on a completely different behavior, such as improved emotion regulation when the target behavior is impulsivity. In addition to the target behaviors hyperactivity and inattention in our study, measured by the SDQ, MPH might affect emotion dysregulation as well, which could be measured by the EDI. GAS is an individualized outcome measure involving goal selection and goal scaling that is standardized in order to calculate the extent to which a patient’s goals are met. Patients and/or their caregivers are allowed to choose their own specific goals in coordination with their treating physician/therapist. This makes GAS a measurement instrument that is very sensitive to change, particularly in small heterogeneous groups.

As the population with ID often presents with atypical side effects, a standardized checklist of side effects of MPH [40] together with an open interview to capture possible atypical side effects will be used to determine (the number of) side effects including sleeping problems.

### Study population

The study population consists of children or adults from the Netherlands with SMS and an ADHD diagnosis established by a multidisciplinary team. Inclusion criteria are a minimum of six years old, a genetically confirmed diagnosis of SMS, and the availability of a caregiver for proxy-reports. Baseline characteristics will be recorded in detail, including age, gender, genetic test results, comorbidity, and medication. Exclusion criteria include presence of a contra-indication for MPH, planned general anesthesia, pregnancy, breastfeeding, current treatment with biologically interfering drugs, substance or alcohol abuse, and incapacity to swallow tablets. The latter may however bias the sample toward a higher functioning segment of SMS. We aim to conduct a patient-centered trial, allowing for a natural setting and flexibility, including the continuation of concurrent therapies such as (for example) sleep medication. Use of concurrent therapies will be recorded.

### Sample size

The sample size calculation was based on a summary measures analysis of the treatment effect as measured with the primary outcome SDQ [41]. The difference between the mean SDQ hyperactivity/inattention ratings in MPH periods and placebo periods was used as a summary measure for the treatment effect in an individual subject. The estimated standard deviation (SD) of 2.3 points for single ratings was used based on a reported standard error for the parent-rated SDQ subscale [42]. Using a test–retest intraclass correlation coefficient (ICC) of 0.84 [43], we decomposed a SD into a within-subject SD of 0.92 and a between-subject SD of 2.11. Assuming an SD of 1 point for the treatment effect, 95% of the subject-specific treatment effects roughly falls within a range of 4 points. Based on the estimate assuming three cycles with seven daily SDQ ratings within each period, a total of 6 subjects will yield 80% power to detect a mean difference of 1.5 points between intervention and placebo periods when assuming a two-sided significance level of 5%.

### Recruitment

Study subjects will be recruited through the two national Dutch SMS multidisciplinary outpatient clinics of ‘s Heeren Loo, and the Dutch SMS patient advocacy organization.

### Trial procedure and study setting

Prior to the start of the trials, the participant and substitute decision maker(s) will have a clinical visit to discuss the procedure in detail and sign the informed consent. Personalized goals with regard to GAS and the PQ and target symptoms will be identified together by the parents and/or primary caregivers, the treating physician, psychologist and/or behavioural therapist, and investigator. During the clinical visit, it will be emphasized that assessors should rate the global effect over the day and should be aware of the possible rebound effect of MPH. The study will be carried out at participants’ home setting and schools or daytime centres if applicable.

**Fig. 2:**
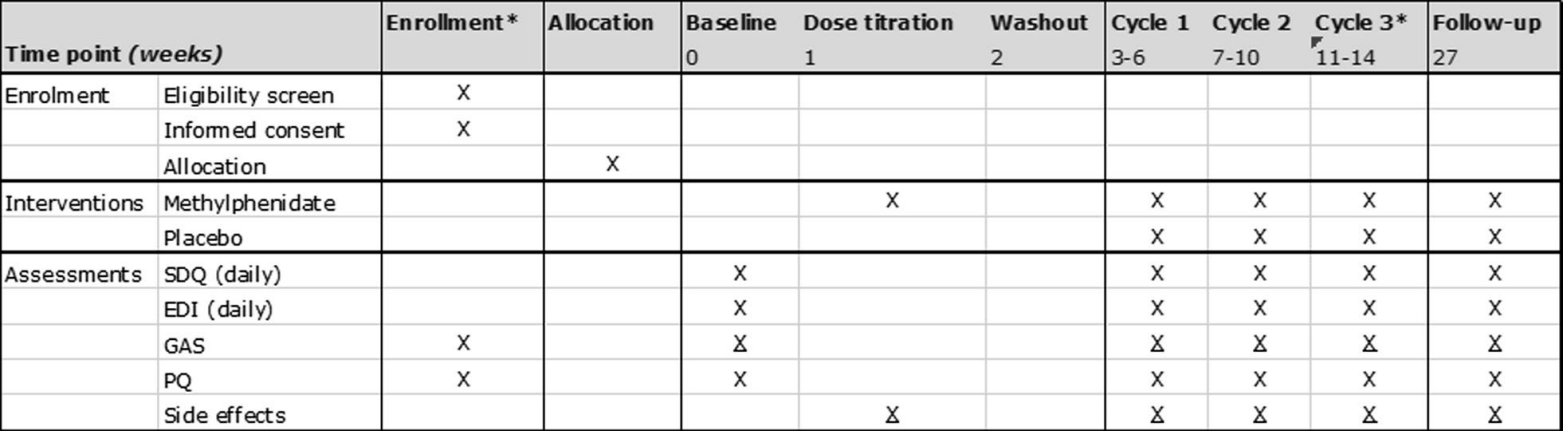
Time schedule of enrolment, interventions, and assessments. Underlined crosses (X) indicate assessments via phone calls. Asterisks (*) indicate the moment with a clinical visit. *EDI* Emotion Dysregulation Inventory, *GAS* Goal Attainment Scaling, *PQ* personal questionnaire, *SDQ* Strengths and Difficulties Questionnaire

The trial will start with a baseline period of seven days without any intervention. A dose titration phase of six days is followed by a washout period of eight days. The individual N-of-1 trial will consist of three cycles each containing four seven-day periods: one active treatment (A), one placebo treatment (B), and two ‘washout’ periods following A and B. The order in which patients receive active and placebo treatment is randomized within each cycle. The medication will be administered at home and/or at school or daytime activities by parents or primary caregivers. During the baseline period and three cycles, the SDQ and EDI will be filled out daily at the end of the day using app-based questionnaires by primary caregivers (Fig. 2). Filling out the questionnaires will take about 1 min a day. At the end of each seven-day period, the investigator will interview patients and/or primary caregivers by phone to evaluate goals [33], to assess possible side effects, to note the general moments that the interventional effects seem to wear off, and to note the perceived treatment received (MPH or placebo). The time expected to complete this interview is 15 min. Each period will include a weekend such that parents can provide assessments of complete days. At the end of the trial period, the participant will have a second and final clinical visit to evaluate the symptoms and study. In consultation with the treating physician, patients may continue with MPH treatment, whether or not at a different dosage. Three months after terminating the N-of-1 trial, another contact moment will take place for a follow-up measurement in which the questionnaires will be filled out and the goals and items of GAS and PQ will be discussed again. To reduce burden as much as possible, assessments solely occur by phone calls apart from the two study visits. The total duration of the trial will be 14 weeks with the additional follow-up measurement after three months.

#### Blinding, treatment allocation, randomization

Participants, parents, caregivers, supervisors of daily activities, clinicians and researchers will all be blinded during the N-of-1 trial. The random allocation sequence will be generated and implemented by the hospital pharmacist for block randomization in a 1:1 ratio and sequentially numbered packages. Participants and the treating physician will be deblinded after completing the three cycles or in case of serious adverse events (SAEs). Investigators involved in data analysis will remain blinded until the end of the follow-up period.

#### Multi-site training plan

A pre-study training meeting will be planned to train clinical investigators and clinical evaluators on study procedures and GAS with a secondary goal to promote reliability of GAS. All clinical and research staff that is involved in either identification or assessment of goals by GAS will be trained by a GAS expert to promote data quality.

### Interventions and dosing schedule

One dose titration kit and a trial kit including MPH (regular tablet) and placebo will be developed and distributed by the Amsterdam UMC hospital pharmacist.

#### Dose titration phase

The MPH dosage will be titrated to achieve the maximum dosage with minimal side effects determined by the psychiatrist or ID physician. Titration dosage will be blinded to the participants and caregivers and comprise two days each of three escalating doses in steps of 2.5 mg of MPH with a total of six days followed by a washout period of at least one week. The individually determined starting dose for the dose titration phase will be based on age and body weight. During the dose titration phase, participants will daily fill out the checklist of side effects of MPH [40]. MPH effectiveness will explicitly not be examined during the titration phase to prevent high dropout rates when participants might get prematurely convinced about the effectiveness.

#### Trial

During the N-of-1 trial, MPH dosage as determined by titration phase or placebo will be administered by caregivers twice daily during breakfast and during lunch (around 7.30 am and 12.30 pm). During washout periods, the placebo will be administered.

#### Follow-up

After the final cycle and unblinding, the participant’s substitute decision maker(s) and clinician will decide on further continuation of MPH treatment before the follow-up measurement. Although a dose titration phase precedes the trial to have a fixed dosage during the N-of-1 trial, participants can switch from dosage or discontinue with MPH in consultation with the treating physician in the follow-up period.

### Safety evaluation

Subjects can leave the study at any time for any reason. The investigator may decide to withdraw a subject from the study for urgent medical reasons. Reasons may include occurrence of treatment-related SAEs or suspected unexpected serious adverse reaction (SUSAR), deterioration of symptoms that require a treatment other than the medication of the trial, and a sudden and acute medical condition related or unrelated to SMS that may interfere with the study. Any sign that indicates resistance among children and mentally incompetent participants, which is defined and discussed with parents and caregivers in advance, will lead to discontinuation of the trial. Completed cycles before withdrawal of a participant will still be analysed. In case of drop-out, a new participant that meets the inclusion criteria will be recruited with a newly randomized sequence. The sponsor will suspend the study if there is sufficient ground that continuation of the study will jeopardise subject health or safety.

Monitoring will be conducted by independent qualified monitors from the Clinical Monitoring Center (CMC). All adverse events (AEs) will be monitored and followed until they have abated, or until a stable situation has been reached. Depending on the event, follow-up may require additional tests or medical procedures as indicated, and/or referral to the general practitioner or a medical specialist.

### Data collection and management

All data will be collected and handled in accordance with the EU General Data Protection Regulation, the Dutch Act on Implementation of the General Data Protection Regulation and Amsterdam UMC standard operating procedures. The Case Report Forms (CRFs) and trial specific documents held by the researcher will be stored securely with access restricted and limited to nominated research staff recorded on the delegation log. A data sharing agreement between Amsterdam UMC and ‘s Heeren Loo will manage additional access for investigators.

The CRFs will be set up in Castor Electronic Data Capture (EDC) in which weekly assessments will be entered. Questionnaires can be filled out digitally using the m-Path app on smartphones [44], on computers (Castor EDC) or by using paper forms. Data from the app will be collected at the end of each trial and will be loaded into Castor EDC. In advance, participants will be recommended to download the m-Path app to easily and confidentially answer the daily questionnaires, although the use of different ways is allowed to enlarge feasibility for raters. For the sake of participant retention, automatic reminders will be sent to raters when questionnaires have not yet been filled in. Participant burden will be limited as much as possible by having contact moments by video-conference or phone instead of a visit. The investigator can also decide to withdraw a subject for urgent medical reasons. A participant who withdraws consent for an assessment of one outcome may be willing to continue with assessments for other outcomes.

A subject identification code list will be used with unique participant identifiers not deducible to patients. Only two investigators will have access to the key. In addition, two methodologists and biostatisticians will have access to the source data for methodological and statistical purposes. Data will be stored for 15 years according to the Amsterdam UMC regulations.

### Statistical methods

An individual treatment effect for each participant will be determined based on summary statistics. A mixed model analysis will be applied for analysing the effectiveness of the intervention at the population level combining data from the individual N-of-1 trials.

The mean treatment effect on the primary outcome will be estimated and tested for significance using a linear mixed model with a fixed effect for treatment (MPH or placebo) and random effects for patient, cycle within patient, and treatment (within patient). The mixed model will account for between-subjects heterogeneity in treatment effect through inclusion of the random treatment effect. Small amounts of missing data will not pose problems for the mixed model analysis because of the many data points per period, assuming data is missing at random. If issues such as singularity arise due to complexity of the models, an analysis based on a summary measure will be performed. A similar method will be used for estimating treatment effects on secondary study parameters. A two-sided significance level of 5% will be used. Analyses will be performed in R, using the lmer package.

## Discussion

To date, research on the efficacy of treatment strategies for behavioural aspects of SMS has been limited. In this N-of-1 series of randomized, placebo-controlled, double-blind multiple crossover trials in patients with SMS and ADHD, the effectiveness of MPH for ADHD symptoms will be examined, including personalized goals as additional outcomes.

N-of-1 studies provide a powerful alternative to larger RCTs, but are still only sporadically reported in rare genetic neurodevelopmental disorders [45]. Debate is still ongoing to what extent an N-of-1 study represents medical research or is part of evidence-based clinical care [46–48]. For instance, for some practitioners starting MPH treatment, blinded crossover periods, the use of placebo and filling out questionnaires is already part of standard care. To provide evidence-based treatment decisions and to prevent polypharmacy, N-of-1 studies might be considered as a much-needed part of clinical care especially in complex patient populations such as individuals with SMs.

Combining personalized and relevant treatment targets while pursuing optimal generalizability is challenging in heterogeneous patient populations such as SMs. Because SMs is accompanied by various and often variable levels of ID and comorbidities, clear diagnostic and eligibility criteria are necessary and baseline characteristics, concurrent therapies, comorbid conditions and target symptoms will be clearly defined to optimize interpretation and generalizability. Also, we will elaborate on setting and location as assessments will be in the participant’s natural environment.

Regarding this symptomatic pharmacological intervention, we chose to add a baseline period. This period allows us to observe the behavior in a non-clinical trial setting and to take the natural course of ADHD symptoms into account. Moreover, to ensure optimal efficacy, tolerability and hence compliance, the highest dosage without side effects will be chosen based on the dose titration phase.

As for the design, the number of participants and crossover periods to detect a clinically relevant treatment effect was selected based on a power analysis, providing the first properly powered N-of-1 study in a rare genetic neurodevelopmental disorder [41]. These are needed when intending to provide estimates of the treatment effect at a population level. Duration of periods was based on the pharmacokinetics and -dynamics of MPH. Although no washout period would suffice pharmacologically, one-week washouts were chosen to account for prolonged psychological effects and for planning purposes.

To pursue optimal generalizability to the entire SMS population, it is of great importance that outcome measures are validated for the patient population and sensitive to change. Multiple data points per period will be acquired to enable estimation of between and within-period variances. To increase the study’s validity, each interventional period includes at least five measurements of the target symptoms, by using the subscale of the SDQ [26, 49]. Several other domains of measurement were chosen, such as sleep quality and personalized measurements. GAS also allows for capturing goals in reduction of caregiver stress, as reduction in symptoms may have benefit for family as well. The EDI will also serve as a generalization measure to evaluate transfer effects of the intervention to a broader domain of functioning. Generalization measures are dependent variables that are taken in addition to the target behavior that are used to evaluate whether an intervention generalizes to other behaviors or settings [39]. A shortened version and a subscale of two outcome measures were selected to minimize assessor’s burden.

Personalized outcome measures such as GAS and the PQ were chosen to appraise subjective experiences in daily life, enabling quantitative expression of meaningful subjective patient experiences while translating these into evidence [50]. Trials tailored to participants by using personalized outcomes may improve treatment adherence as well. Although GAS has not yet been validated and performed in N-of-1 designs nor as an outcome measure in rare genetic disorders with ID, it may be a valuable tool in a complex and heterogeneous population such as SMS. This study will introduce GAS in the N-of-1 design and might be a step towards validation of this personalized outcome measure in rare disorders.

Regarding the analysis, a mixed model analysis was selected to analyze the effectiveness of the intervention at the population level, accounting for between-subjects heterogeneity. Ancillary analyses will be performed to evaluate period effects and intrasubject correlation.

Limited burden is expected and maximal relevance and treatment adherence is ensured, as an N-of-1 study provides the unique opportunity to tailor interventions and outcomes to individual patients. To optimize compliance, daily questionnaires will be filled out using a user-friendly app and contact moments will mainly take place via digital or telephone calls. Caregivers may experience some burden because of longer withholding of active medication due to one-week washouts to account for eventual psychological effects; this was also the main aberrance from clinical care, necessitating institutional review board (IRB)-approval. On the other hand, every participant is exposed to the active treatment condition and the effect of the individual treatment will be assessed in the best available way, minimizing placebo effects, observer effects, and confirmation biases. After the N-of-1 trial, participants and their representative(s) will be fully informed on the effectiveness of the intervention, allowing shared decision making on future treatment. Participants might thus be particularly motivated to participate in an N-of-1 study due to the existing paucity of evidence and the fact that all subjects will receive an evidence-based assessment of their individual treatment effect.

## Conclusion

This N-of-1 study will allow the delivery of personalized care while acquiring evidence of MPH for ADHD in the SMS population. We expect that use of the N-of-1 methodology and patient-centered outcome measures will assist in realizing the urgently needed evidence-based interventions in patients with rare genetic neurodevelopmental disorders. This protocol will be applicable for other genetic syndromes, and more N-of-1 series will allow cross-disorder comparisons and investigation of generalizability to the whole population with these disorders and/or ID. This study protocol can be used as a model to empower other clinician-researchers to investigate much-needed symptomatic pharmacological as well as disease-modifying interventions in rare disorders using a collaborative and multi-disciplinary approach.

## Data Availability

Not applicable.
